# Harnessing Copper Nanoparticles for Antimicrobial Applications: Advances and Challenges

**DOI:** 10.3390/antibiotics14111170

**Published:** 2025-11-20

**Authors:** Diogo S. Pellosi, Giovanna S. M. Paiva, Vitor G. Vital, Adriano L. Mendes, Nubia G. Santos, Fernanda K. Kuriki, Keith D. L. Lira, Giovana C. M. Oliveira, Yasmin R. Gomes, Flavia G. Lobo, Vinicius T. Santos, Marcio R. Silva, Ricardo A. G. Silva, Suzan P. Vasconcellos

**Affiliations:** 1 Chemistry Department, Federal University of Paraná (UFPR), Curitiba 81531-980, PR, Brazil; diogo.pellosi@ufpr.br; 2 Institute of Environmental, Chemical and Pharmaceutical Sciences—ICAQF, Federal University of São Paulo (UNIFESP), Diadema 09913-030, SP, Brazil; giovanna.paiva@unifesp.br (G.S.M.P.); vitor.vital@unifesp.br (V.G.V.); adriano.mendes15@unifesp.br (A.L.M.); ngsantos@unifesp.br (N.G.S.); fernanda.kaori@unifesp.br (F.K.K.); keith.lira@unifesp.br (K.D.L.L.); yasmin.rodrigues30@unifesp.br (Y.R.G.); galdino.ricardo@unifesp.br (R.A.G.S.); 3 Institute of Science and Technology—ICT, Federal University of São Paulo (UNIFESP), São José dos Campos 12231-280, SP, Brazil; giovana.correia@unifesp.br; 4 Department of Research and Development, Termomecanica São Paulo S.A., São Bernardo do Campo 09612-000, SP, Brazil; flavia.lobo@termomecanica.com.br (F.G.L.); vinicius.santos@termomecanica.com.br (V.T.S.); marcio.rodrigues@termomecanica.com.br (M.R.S.)

**Keywords:** copper nanoparticles, antimicrobial resistance, nanotechnology, cytotoxicity, ecotoxicology, biomedical

## Abstract

Antimicrobial resistance (AMR) is one of the most significant global health threats of the 21st century, driving the urgent search for alternatives to conventional antibiotics. Copper nanoparticles (CuNPs) have gained attention due to their broad antimicrobial spectrum, cost-effectiveness, and versatile applications in medicine, agriculture, and the food industry. This review provides a systematic overview of the advances in CuNP synthesis, mechanisms of antimicrobial action, biomedical and industrial applications, and associated toxicity issues. A comprehensive literature review was conducted, covering chemical, physical, and biological synthesis strategies; mechanistic studies on microbial inhibition; and experimental reports on biomedical and environmental applications. A comparative analysis revealed opportunities, limitations, and knowledge gaps, with particular emphasis on cytotoxic and ecotoxicological aspects. CuNPs show strong antimicrobial activity against bacteria, fungi, viruses, and multidrug-resistant strains through mechanisms such as reactive oxygen species (ROS) generation, membrane disruption, and DNA/protein interactions. Their use in medical devices, wound dressings, textiles, and packaging materials underlines their application potential. However, cytotoxicity to mammalian cells, ecological risks, and the lack of standardized safety protocols remain critical challenges. Particle size, morphology, and surface chemistry strongly influence both efficacy and toxicity, underlining the importance of controlled synthesis and functionalization. Overall, CuNPs represent a promising strategy to tackle the AMR crisis. Future research should focus on environmentally friendly and surface-modified synthesis approaches, standardized toxicity assessments, and robust regulatory frameworks. By balancing antimicrobial efficacy with biosafety and sustainability, CuNPs could become a transformative platform for clinical, industrial, and environmental applications.

## 1. Introduction

Microbial infections have been treated with antimicrobial drugs for decades; however, the rising microbial resistance against the known drugs has been a challenge that needs to be addressed for human health. Overall, microbial resistance is the mechanism that microorganisms have developed to survive when faced with antibiotics. In this scenario, the need for new drugs or strategies to overcome microbial resistance is urgent, as it represents a global health threat [1].

In this sense, this review examines recent advances in the use of copper nanoparticles (CuNPs) as antimicrobial agents, with a focus on their potential to address the global challenge of antimicrobial resistance. This study presents a systematic literature review aimed at discussing new findings and contradictions regarding copper nanoparticles. The following databases were used for the assessment: Google Scholar, Web of Science, ScienceDirect, and PubMed. The search covered publications from 2005 to 2025. The following keywords were employed: “copper nanoparticles,” “antimicrobial applications,” “antimicrobial resistance,” “nanotechnology,” “copper chemical synthesis,” “metallic nanoparticle green synthesis,” “biosynthesis,” “biological synthesis,” “physical methods,” “properties,” “toxicity,” “copper environmental impacts,” and “antimicrobial activity.” Only English language manuscripts were adopted as bibliographic references. It is important to mention that duplicated references and conference abstracts were excluded, and only referenced articles were fully analyzed.

Here, we outline synthesis approaches, physicochemical properties, and key mechanisms of antimicrobial action, emphasizing their efficacy against bacteria, fungi, viruses, and multidrug-resistant strains. Applications in biomedical fields, such as coatings, wound dressings, and textiles, are emphasized, as well as concerns regarding cytotoxicity and environmental impact. By integrating the current state of knowledge, this review aims to highlight the opportunities and challenges of CuNPs and identify future research directions.

### 1.1. Overview of the Global Challenge of Antimicrobial Resistance

Antimicrobial resistance (AMR) is considered one of the greatest global health threats of the 21st century. In 2019, it was associated with nearly 5 million deaths worldwide, of which 1.27 million were directly attributable to AMR—numbers that exceed the annual mortality caused by HIV/AIDS or malaria [2,3]. In the current scenario, projections indicate that between 2025 and 2050, the number of deaths associated with AMR will reach around 169 million. These data highlight the urgency of developing new approaches to overcome this situation [4].

AMR refers to the ability of microorganisms, including bacteria, viruses, and fungi, to develop resistance to antimicrobial drugs designed to eliminate or inhibit them. This complicates the treatment of infectious diseases and has profound social and economic implications [5,6]. World Bank projections suggest that, if left unchecked, AMR could reduce global GDP by around 4% by 2050, pushing millions of people into poverty and exacerbating challenges in healthcare, food security, and livestock production [7]. The cross-cutting nature of AMR underscores the need for integrated approaches such as the “One Health” perspective, which emphasizes the interconnectedness between human, animal, and environmental health [7].

Of particular concern are multidrug-resistant bacterial groups such as the ESKAPEE pathogens (*Enterococcus* sp., *Staphylococcus aureus*, *Klebsiella pneumoniae*, *Acinetobacter baumannii*, *Pseudomonas aeruginosa*, *Enterobacter* sp., and *Escherichia coli*). These organisms are frequently associated with persistent infections—including urinary tract infections, sexually transmitted infections, and nosocomial infections—and they are increasingly resistant to conventional treatments. The indiscriminate use of antibiotics during the COVID-19 pandemic has further accelerated the development of resistant strains and exacerbated the global AMR crisis. Regions with poor access to drinking water, quality healthcare, and adequate sanitation are disproportionately affected and account for nearly 90% of AMR-related deaths [8,9].

The emergence of resistant and multidrug-resistant bacteria is largely a consequence of the overuse of antibiotics and improper disposal of drugs that promote genetic diversification and horizontal transmission of resistance genes. This phenomenon leads to escalating healthcare costs, increased morbidity and mortality, and an urgent need for alternative antimicrobial strategies [10]. In recent years, metal nanoparticles (MNPs) have emerged as promising candidates to address these challenges due to their unique physicochemical properties and diverse antimicrobial mechanisms. Among them, copper nanoparticles show particular promise due to their broad spectrum of activity and ability to bypass conventional resistance pathways [11,12]. They offer a new approach to combating super-resistant bacteria by targeting structural and metabolic damage to the cell as a whole, rather than acting solely on specific cellular mechanisms, as is characteristic of traditional antibiotic compounds. This multivalent mode of action can help to delay the emergence of new microbial resistances, mainly due to the inherently multi-target nature of nanoparticles [13]. It is important to mention that nanoparticles can act not only independently but also in synergy with antibiotics, improving drug efficacy and disrupting resistance mechanisms, such as biofilm formation [14].

### 1.2. Historical Use of Copper as an Antimicrobial Agent

Copper is already known for its antimicrobial properties. Since ancient periods, it has been one of the first materials used in its native form [15,16]. As early as before 2600 BC, the Egyptians described the use of copper to purify water and treat wounds, infections, and ulcers. The Romans also described the use of copper for medicinal purposes. More recently, during the cholera outbreaks of the 19th century, workers from the copper industry showed remarkable resistance to this infection [17].

In the modern era, experimental studies have repeatedly confirmed the antimicrobial efficacy of copper. Majno [18] attempted to reproduce the copper-based compounds described in Egyptian texts, while researchers at the University of Southampton in the 1990s demonstrated that bacteria such as *Escherichia coli* O157:H17 and *Listeria monocytogenes* were killed quickly on copper surfaces but survived for longer periods on stainless steel [19,20,21]. These results are promising, as the *Escherichia coli* O157:H17 is capable of surviving in complex environments during extended periods of time, especially at low temperatures [20]. Furthermore, in assays with *Listeria monocytogenes*, a high concentration of bacteria was used to represent a “worst-case” scenario, and survival was significantly lower in the copper-based alloy compared to stainless steel, with viable cells still being detected on stainless steel after 24 h [21].

Later studies confirmed the effectiveness of copper and copper-based alloys against antibiotic-resistant pathogens such as MRSA (methicillin-resistant *Staphylococcus aureus*) and VRE (vancomycin-resistant *Enterococcus*), which were killed within hours, even at low temperatures [17,22,23]. Some reports have shown that, in contrast to stainless steel at 22 °C, copper is intrinsically antibacterial against MRSA, EMRSA-1, and EMRSA-16 and can prevent their long-term persistence [19]. The obtained results reinforce the applicability of copper-coated surfaces in various environments, especially in hospital settings, ensuring a reduction in the proliferation of bacteria and infections.

In addition, concerns over the transmission of pathogens via soft surfaces in healthcare settings have spurred the development of copper-impregnated textiles and polymers through nanotechnology. These copper-based materials exhibit antimicrobial activity against bacteria, fungi, and viruses, including HIV-1 and West Nile virus, thus reducing the risk of healthcare-associated infections [17,24].

Copper has also played an important role in agriculture. The Bordeaux mixture, developed by Pierre-Marie Alexis Millardet in 1885, used copper sulfate to control *Plasmopara viticola* in grapevines, becoming the first widely used fungicide lamp [25]. Today, copper nanoparticles are being investigated as alternatives to conventional copper-based agrochemicals. They offer improved bioavailability and slow release while reducing environmental issues such as accumulation in soil and groundwater. This controlled release improves plant uptake and prolongs efficacy in the soil [26]. All in all, these historical milestones highlight the continuing role of copper as a powerful antimicrobial agent in medicine, public health, and agriculture.

### 1.3. Emergence of Nanotechnology in Antimicrobial Strategies

The increasing threat of AMR, combined with the emergence of novel pathogens, has driven the development of alternative therapies beyond conventional antibiotics. Nanotechnology has emerged as one of the most promising approaches, especially after the COVID-19 pandemic [27], which highlighted the potential of organic, hybrid, and inorganic nanoparticles as microbicidal agents, with metallic nanoparticles—such as silver, gold, zinc, titanium, and copper—standing out due to their broad antimicrobial activity. Among these, copper nanoparticles are of particular interest for this review [27]. When combined with polymers, they can form hybrid nanocomposites with improved efficacy, biocompatibility, and stability, which can be used in coatings for medical devices, hospital surfaces, and textiles [28].

In addition, copper nanoparticles can be used in combination with antibiotics to increase antimicrobial efficacy, mitigate resistance, and minimize adverse effects. Ref. [29] also emphasized the unique physicochemical properties of metal nanoparticles—including surface area to volume ratio, generation of ROS, and direct interaction with microbial membranes—that allow them to disrupt essential proteins, damage DNA, and prevent biofilm formation. These properties provide a versatile means of combating resistant microorganisms [29]. Despite these advantages, cytotoxicity and safety issues remain, highlighting the need for further research to ensure effective and biocompatible applications. Nonetheless, the integration of nanotechnology into antimicrobial strategies represents a crucial step forward in combating AMR and expanding the arsenal against microbial threats.

## 2. Synthesis of Copper Nanoparticles

Copper nanoparticles (CuNPs) can be synthesized by chemical, physical, and biological methods, each of which has its own advantages and limitations. There are two main approaches: top-down and bottom-up systems. The top-down approach allows nanomaterials to be obtained by breaking down a larger portion of the materials to obtain the desired nanostructure (from macro to nano). This is generally used in the areas of microelectronics, engineering, and physics, usually involving physical methods. The bottom-up approach involves the use of the components, initially present at the atomic scale, that self-organize into nanostructured materials. They are nanostructures built either atom by atom, molecule by molecule, or through clusters of atoms or molecules. This is commonly used in chemistry and biology, while chemical methods are generally more widely employed [30,31,32].

**Figure 1:**
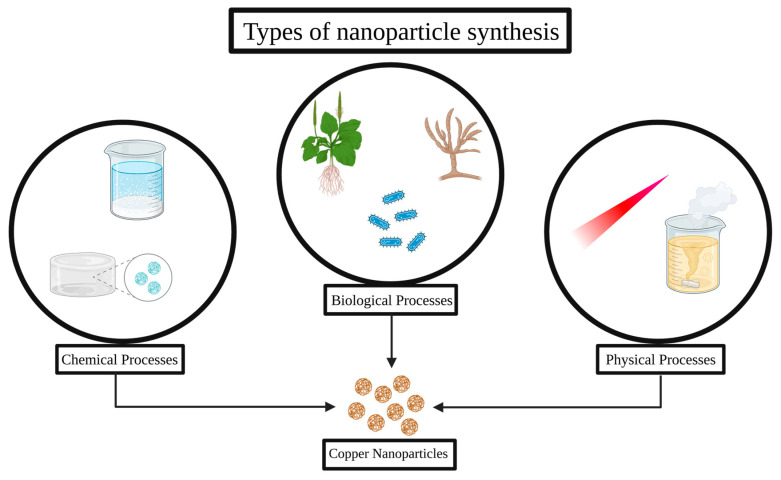
Different approaches for copper nanoparticle synthesis.

Chemical methods are the most common and reliable way to reduce copper salts in solution to produce nanoparticles, typically in the presence of stabilizers or capping agents that prevent agglomeration. Physical methods generally involve the use of high-energy processes, such as laser ablation, evaporation condensation, or ball milling, to produce nanoparticles. These processes often provide good control over particle size and morphology but are usually costly and require sophisticated equipment. Biological synthesis, also known as “green synthesis”, uses plant extracts, microorganisms or microbial extracts, or biopolymers as reducing and stabilizing agents, constituting a more environmentally friendly and cost-effective approach. Figure 1 summarizes the synthetic methods described in the present review.

### 2.1. Chemical Methods

Chemical synthesis is one of the most widely used approaches to produce metallic nanoparticles. It offers advantages in controlling particle size, crystallinity, and morphology, and it is also cost-effective and scalable [33,34]. The most common strategy is chemical reduction, in which copper salts such as CuSO_4_, CuCl_2_, or Cu(NO_3_)_2_ are reduced to elemental copper (Cu^0^) using agents such as sodium borohydride (NaBH_4_), ascorbic acid, hydrazine, or glucose [35,36,37,38]. To prevent aggregation and oxidation, stabilizers such as polyvinylpyrrolidone (PVP), citrate, or polyethylene glycol (PEG) or polysaccharides such as chitosan are usually added [28,39,40]. Another new approach is the use of a thin shell of another oxidation-resistant metallic nanostructure, such as silver or gold, creating stable core–shell structures [40]. The reactions are usually carried out in aqueous or alcoholic solvents at 30–120 °C, sometimes under an inert atmosphere [41]. Although this method is inexpensive and tunable, it requires careful optimization to ensure long-term stability [42,43].

Recent studies have shown that chemical reduction can be customized for biomedical applications. For example, CuNPs synthesized with ascorbic acid and stabilized with PVP showed strong antimicrobial activity against *Pseudomonas aeruginosa* and *Staphylococcus aureus* while exhibiting moderate cytocompatibility with mammalian fibroblasts [44]. Similarly, chitosan-stabilized CuNPs have shown dual activity by acting as both antimicrobial agents and promoters of wound healing due to the intrinsic bioactivity of chitosan [45]. These results suggest that the choice of stabilizer is critical not only for stability but also for defining biological performance.

Electrochemical synthesis is another important approach, in which copper ions are reduced in solution in an electrolytic cell. Copper electrodes immersed in Cu(NO_3_)_2_ or CuSO_4_ solutions release Cu^2+^ at the anode, which is reduced at the cathode to form CuNPs [46,47]. The morphology can be modulated by surfactants or polymers, but the reproducibility depends on the precise control of parameters such as voltage and current density. Electrochemical methods have also been used to deposit CuNPs directly onto biomedical devices to create antimicrobial coatings on catheters and dental implants that significantly reduce bacterial colonization in preclinical models [46,48].

In thermal decomposition, precursors such as copper acetate, copper acetylacetonate, or copper oxalate are heated in high-boiling solvents (150–300 °C), usually under an inert atmosphere [49,50]. This method leads to uniform, highly crystalline particles and enables morphology tuning to nanospheres, nanorods, or nanocubes [51]. However, it requires organic solvents, controlled environments, and a high energy input, making it more complex and costly than solution-based reduction [52]. Despite the higher costs, thermal decomposition has been particularly useful in catalysis, where CuNPs with specific crystal facets (e.g., nanocubes) exhibit superior performance in CO_2_ reduction and organic transformations [53].

Finally, the sol–gel route offers excellent homogeneity and versatility. Here, copper precursors are hydrolyzed and condensed to form a porous gel matrix, which is then calcined or reduced to obtain metallic nanoparticles [54,55]. This method is particularly suitable for embedding CuNPs in ceramic or silicate carriers, but it is relatively slow and involves several steps [56]. Recent applications include the development of CuNP–silica nanocomposites for use in food packaging films with sustained antimicrobial activity, demonstrating practical potential in preventing spoilage and extending the shelf life of perishable goods [57].

### 2.2. Physical Methods

Physical methods rely on mechanical, thermal, or electromagnetic energy to fragment bulk copper or generate nanoparticles from vaporized atoms [43]. In physical vapor deposition (PVD), including thermal evaporation and sputtering, copper is evaporated in a vacuum or in an inert atmosphere, followed by condensation into nanoparticles [58,59]. These methods offer excellent control over size, film thickness, and purity, making them ideal for coatings and electronics; however, they are less suitable for large-scale nanoparticle production [60]. For example, CuNPs deposited by sputtering were integrated into orthopedic implants, where they significantly reduced the formation of biofilms without compromising mechanical integrity. Thermal vaporization was also used to produce thin copper layers with antiviral properties, which were effective against influenza and coronavirus strains under laboratory conditions [61].

Laser ablation is another precise but costly method, in which high-energy pulsed lasers irradiate copper targets immersed in a liquid, forming a plasma jet from which nanoparticles are formed [62,63]. The size and morphology can be fine-tuned by adjusting the laser parameters and the composition of the liquid [64]. This method produces very pure, well-dispersed CuNPs without chemical reagents but suffers from low yields and expensive equipment requirements [65,66]. Interestingly, laser-ablation-derived CuNPs have been used in water disinfection, where their high surface reactivity contributed to rapid inactivation of *E. coli* and *Vibrio cholerae*, highlighting potential for environmental applications [67].

### 2.3. Biological (Green) Synthesis

Biological or “green” synthesis uses plant extracts, fungi, bacteria, or algae as environmentally friendly reducing and stabilizing agents [68]. This approach avoids hazardous reagents, promotes biocompatibility, and is interesting for biomedical applications [69]. Plant-mediated synthesis uses extracts rich in polyphenols, flavonoids, terpenoids, and proteins to reduce Cu^2+^ ions while capping the nanoparticles [70,71]. Although the method is simple, scalable, and inexpensive, the variability of the extract composition limits reproducibility; it depends on the source and type of plant extract, as well as the concentration of phytochemicals, in addition to the extraction method, which influences the characteristics of a nanostructure, potentially resulting in particles with less uniform size distribution and morphology [69,72].

On the other hand, actinomycetes are widely recognized for their generation and many uses of nanoparticles. Their composition, which features greater enzymatic diversity and additional bioactivity, makes them more efficient than other bacteria, especially in facilitating improved control over the shape and size of nanoparticles for large-scale application [73,74,75,76].

In microbial synthesis, bacteria or fungi are used to reduce copper ions either intracellularly via enzymatic activity or extracellularly via secreted metabolites [72,77]. Strains such as *Bacillus subtilis*, *Pseudomonas stutzeri*, and *Fusarium oxysporum* have been successfully used [78,79]. Although this method is highly selective, it requires sterile conditions, optimized growth media, and longer synthesis times [80]. Fungi are particularly promising due to their high secretion of proteins and enzymes. They produce stable, monodisperse nanoparticles and are easier to scale up than bacterial systems [81,82].

It is also important to mention the synthesis process using algae cells. Their composition, including proteins and secondary metabolites, allows them to work like a biofactory, hyperaccumulating metals and transforming them into nanoparticles. Additionally, their good cost-effectiveness and the possibility of large-scale application in nanoparticles of different metals stand out. This process is related to specific released pigments; in the case of copper nanoparticles, brown algae are most commonly used. Like other green methods, the low or non-existent toxicity allows the synthesized nanoparticles to be used more safely in the biomedical field [83,84,85].

### 2.4. Comparison of Synthesis Methods Regarding Particle Size, Morphology, and Stability

Each synthesis route leads to CuNPs with different size ranges, morphologies, and stability profiles [43]. Chemical reduction methods are versatile and produce 10–50 nm sized particles with tunable shapes and good colloidal stability, especially when stabilizers are used [86,87]. However, physical methods such as PVD and laser ablation generally produce smaller (2–20 nm), surfactant-free, and high-purity nanoparticles, which are susceptible to oxidation without a protective coating [88,89]. Biological methods yield slightly larger particles (20–100 nm), which are often spherical and have a natural biomolecular cap that improves stability and biocompatibility but are less reproducible [90]. In addition, several advanced or hybrid synthesis approaches have emerged, including microwave-assisted green synthesis, microfluidic synthesis, and ultrasound-assisted reduction, as well as photochemical, solvothermal, hydrothermal, electrochemical, and plasma-assisted methods, which aim to improve particle uniformity, size control, reproducibility, and process scalability [91,92,93,94].

Overall, chemical methods are characterized by precision and scalability, physical methods are characterized by purity and control, and biological synthesis methods are characterized by sustainability and inherent biocompatibility. Table 1 summarizes the main differences between different synthesis approaches. The optimal method depends on the intended application, whereby performance, costs, reproducibility, and environmental aspects must be weighed up.

### 2.5. Comparison of Copper Nanoparticles and Other Metallic Nanoparticles

Metallic nanoparticles have gained considerable attention due to their unique physicochemical and biological properties. Each of them has specific advantages for diverse applications, such as in the medical field, but also holds certain limitations. Copper nanoparticles are recognized for their antimicrobial and antifungal activity, biomedical applications, wound-healing properties, and use for the prevention of food spoilage when incorporated in food packaging, but there are some limitations [2,44,45,52,96,97,98]. Table 2 presents the advantages and limitations of metallic nanoparticles of copper, zinc oxide, silver, gold, and titanium dioxide.

## 3. Mechanisms of Antimicrobial Action

**Figure 2:**
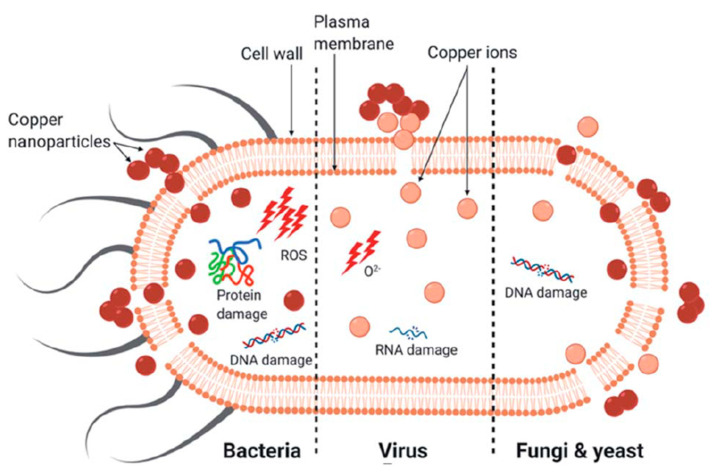
The primary mechanism of death in different microorganisms (bacteria, viruses, fungi, and yeast) by copper nanoparticles.

The antimicrobial efficacy of copper nanoparticles (CuNPs) is based on a combination of physicochemical interactions and biochemical interferences that affect microbial survival. In contrast to conventional antibiotics, which often act through a single molecular target, CuNPs exert a multifaceted mode of action that includes oxidative stress, membrane destabilization, protein, DNA, and RNA damage, interference with enzymatic processes, and the controlled release of copper ions (Figure 2). This multimodal activity not only increases their efficacy against a variety of pathogens but also reduces the likelihood of resistance development. The following subsections summarize the main mechanisms described in the literature and highlight both their molecular basis and their practical implications for antimicrobial applications.

### 3.1. Generation of Reactive Oxygen Species (ROS)

The antimicrobial activity of copper nanoparticles (CuNPs) is closely linked to their ability to generate reactive oxygen species (ROS), which trigger oxidative stress in microbial cells [107]. Once they come into contact with cells, released copper ions catalyze ROS production, leading to lipid peroxidation, protein denaturation, and DNA damage [108]. Interestingly, some biologically synthesized CuNPs exhibit both antimicrobial and antioxidant properties, as observed in CuNPs extracted from *Stenotrophomonas rhizophila*, which showed 48–94% antioxidant activity due to phenolic cap molecules acting as chelators and free radical scavengers [109]. Further studies have confirmed that ROS-mediated antimicrobial effects are not only bactericidal but also fungicidal. For example, CuNPs, synthesized from plant extracts, suppressed the growth of *Candida albicans* by inducing intracellular ROS accumulation, leading to apoptosis-like cell death. In addition, the ROS burst generated by CuNPs has been associated with virus inactivation, as in the case of copper oxide nanoparticles destroying influenza A virus particles by lipid peroxidation and protein cross-linking [110].

An important trend is the design of specialized nanoparticles for autonomous ROS liberation, independent of ion release, to minimize the risk of microbial adaptation. For example, copper nanoclusters synthesized from Cu(NO_3_)_2_, cysteine, and chitosan produced hydroxyl radicals (-OH) and hydrogen peroxide (H_2_O_2_) under visible light but maintained their bactericidal activity even without irradiation when concentrated at 0.5 mM [111]. Similarly, bimetallic Cu/Zn nanoparticles with surface-deficient cocrystals, at concentrations of 256 µg/mL, facilitated O_2_ adsorption and electron transfer by mimicking the activity of superoxide dismutase and increasing intracellular ROS accumulation [111]. Since ROS generation is also a natural mechanism of immune defense [112], exploiting this pathway with CuNPs represents a promising strategy to combat multidrug-resistant pathogens. Interestingly, photocatalytic composites based on CuO/TiO_2_ have also been investigated, in which visible light enhances the antimicrobial effect through plasmonically supported ROS formation. These hybrid nanostructures offer a route to the development of multifunctional antimicrobial coatings for hospital surfaces [113].

Beyond ion-mediated pathways, visible light can activate plasmonic copper nanostructures (Cu^0^, Cu/Cu_2_O, or Cu-based bimetals) via localized surface plasmon resonance (LSPR) by concentrating the electromagnetic field on the surface of the nanoparticles and promoting hot charge carriers that are transferred to O_2_/H_2_O to generate -OH, O_2_^−^, and H_2_O_2_ [113,114]. This SPR-enhanced ROS generation under visible light has been demonstrated for plasmonic CuNPs and other plasmonic NPs (e.g., Ag and Au), which increase antimicrobial efficacy without external oxidizing agents and at low irradiances [115].

### 3.2. Disruption of Microbial Cell Membranes

Another well-documented mechanism is the direct interaction of CuNPs with microbial membranes. Due to their positive surface charge, CuNPs bind electrostatically to negatively charged bacterial surfaces and induce a disorganization of the lipids, formation of pores, and disruption of the structures [115]. This effect is particularly evident in Gram-negative bacteria, where CuNPs destabilize lipopolysaccharides, and in Gram-positive bacteria, where teichoic acids are disrupted, facilitating nanoparticle penetration [116]. For example, CuNPs synthesized with aloe vera extract have been shown to cause pore formation in the membranes of *Escherichia coli* within 30 min of exposure, leading to leakage of proteins and nucleotides. In fungal pathogens, CuNPs interact with ergosterol-rich membranes, as shown in *Candida tropicalis*, where nanoparticles caused leakage of cytoplasm and severe morphological deformations [117].

Released Cu^2+^ ions further impair membrane potential by binding to phosphate and sulfhydryl groups, leading to depolarization, cytoplasmic leakage, and metabolic collapse [118]. Morphology plays a crucial role in *S. aureus* inhibition, with copper nanorods with sharp ends showing up to 3.4-fold higher efficacy in membrane rupture than spherical nanoparticles [116]. Scanning electron microscopy confirms that high concentrations of CuNP (1000 µM) produce discontinuities in *E. coli* membranes, an effect that does not occur with ionic copper alone [115]. In biomedical contexts, wound dressings impregnated with CuNPs have demonstrated rapid inhibition of *E. coli* and *S. aureus* colonization by direct membrane disruption, accelerating wound closure in animal models [119].

These principles can be transferred to biomedical applications. Catheters coated with CuNP suppressed *Candida auris* biofilms by 89%, and copper nanocomposites in tissues inactivated 99.9% of SARS-CoV-2 within four hours by disrupting the viral lipid envelope [115,116]. Nevertheless, the similarity of these mechanisms to those acting on mammalian membranes underscores the need to balance antimicrobial efficacy and biocompatibility [118].

### 3.3. Interaction with Microbial DNA and Proteins

CuNPs also impair genetic and enzymatic functions. ROS generated by CuNPs oxidize nitrogenous bases and induce DNA strand breaks, which impair replication and transcription. In *E. coli*, exposure led to degradation of 80% of plasmid DNA within two hours, while Cu^2+^ ions directly complexed with phosphate groups, distorted the double helix, and inhibited DNA polymerase [119,120]. In *S. aureus*, CuNPs downregulated DNA repair genes (recA and uvrA) and thus increased mutation susceptibility [121]. Similarly, CuNPs functionalized with chitosan caused oxidative lesions in the DNA of *Klebsiella pneumoniae*, as confirmed by the comet assay, and led to downregulation of SOS response genes, suggesting their potential use against multidrug-resistant strains [122].

Protein inactivation occurs through the binding of copper to sulfhydryl groups (-SH), which denatures important enzymes. In *P. aeruginosa*, catalase activity decreased by 70% after CuNP exposure, reducing the ROS detoxification capacity of the bacterium [123]. Proteomic shifts included suppression of porins and transport proteins, disrupting homeostasis [120]. In *Candida albicans*, CuNPs disrupted ribosomal proteins and stopped protein synthesis [124].

The so-called “Trojan horse” mechanism, in which intact CuNPs enter the cells via porins or endocytosis, releases Cu^2+^ directly into the cytoplasm. In *Salmonella enterica*, 65% of CuNPs localized to the nucleus within one hour, which correlated with DNA fragmentation [125]. Such combined DNA and protein damage supports the broad-spectrum activity of CuNPs but also emphasizes the importance of controlled dosing to reduce selection pressure. However, the extent to which this intracellular accumulation occurs under realistic biological conditions remains uncertain, as experimental setups often involve higher nanoparticle concentrations or simplified in vitro systems.

Overall, while the multifaceted antimicrobial mechanisms of CuNPs underscore their therapeutic potential, the variability in experimental outcomes highlights the necessity of standardized testing and mechanistic clarification to ensure both efficacy and biosafety in clinical and environmental applications.

### 3.4. Release of Copper Ions and Their Role in Microbial Inhibition

The antimicrobial efficacy of CuNPs is also mediated by the gradual release of Cu^+^ and Cu^2+^ ions, enhanced by the high surface area of nanoparticles [126]. These ions compromise membrane integrity, increase permeability, and trigger cytoplasmic leakage [127]. They also participate in Fenton-like reactions, generating hydroxyl radicals that damage DNA, lipids, and proteins [128]. In practical applications, this controlled ion release has been explored in food preservation, where CuNP-embedded films extended the shelf life of tomatoes by reducing bacterial spoilage through continuous Cu^2+^ ion diffusion [129].

Copper ions can substitute essential cofactors such as iron and zinc in metalloenzymes, impairing catalysis and leading to metabolic collapse [130]. Recent findings describe “cuproptosis,” a copper-induced cell death mechanism involving binding of Cu^2+^ to lipoylated proteins in the tricarboxylic acid cycle, resulting in proteotoxic stress [131]. This concept has sparked interest in oncology, where CuNP-mediated cuproptosis is being investigated as a therapeutic avenue to selectively induce death in tumor cells resistant to apoptosis [132].

The ion release rate depends on nanoparticle composition, morphology, pH, and exposure time. Both metallic (Cu^0^) and oxide-based (CuO, Cu_2_O) nanoparticles display strong antimicrobial activity against bacterial and fungal strains [133,134,135,136]. Thus, controlled release of copper ions is central to the sustained antimicrobial action of CuNPs.

## 4. Spectrum of Antimicrobial Activity

The antimicrobial spectrum of copper nanoparticles (CuNPs) includes bacteria, fungi, and viruses, making them versatile agents for clinical and environmental applications. Their multimodal mechanisms of action give them activity on different microbial groups and limit the risk of resistance compared to conventional antibiotics. Studies have shown that they are effective not only against planktonic cells but also against biofilms, spores, and multidrug-resistant (MDR) pathogens, which remain among the most difficult targets in the fight against infectious diseases. Beyond human health, the broad spectrum of CuNPs opens up opportunities for applications in agriculture, food preservation, and water treatment, underlining their translational potential.

### 4.1. Efficacy Against Gram-Positive and Gram-Negative Bacteria

**Figure 3:**
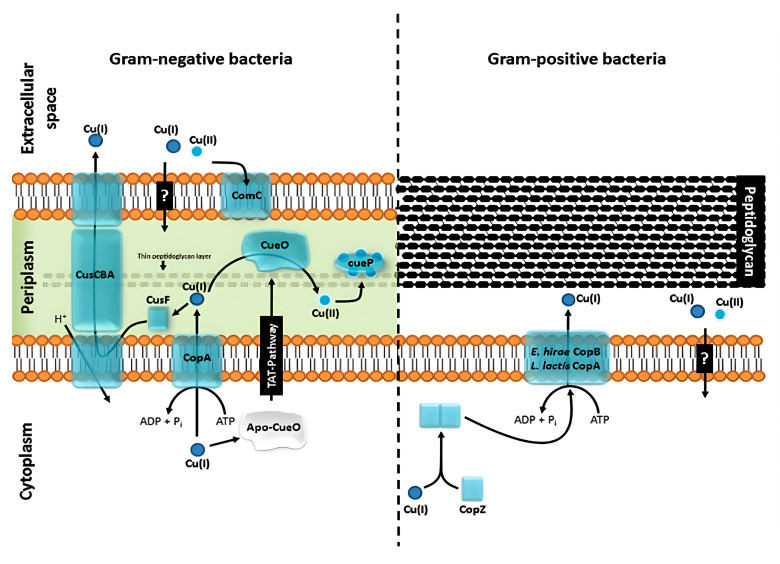
Schematic illustration of the copper homeostasis systems of a model Gram-negative organism (*Salmonella enterica* sv. *Typhimurium* (**left**)) and a model Gram-positive organism (*Staphylococcus aureus* ATCC 12600 (**right**)). In Gram-negative organisms (**left**), the CusCBA system extrudes Cu(I) from the periplasm, aided by the chaperone CusF. CopA transports Cu(I) from the cytoplasm to the periplasm, while CueO oxidizes Cu(I) to Cu(II) and CueP acts as a periplasmic reservoir. The question mark indicates steps that are not yet fully elucidated, especially those related to the pathway of Cu(I)/Cu(II) entry through the outer membrane. In Gram-positive bacteria (**right**), the chaperone CopZ delivers Cu(I) to the ATPase P1B (CopA/CopB), which is responsible for its extrusion. The second question mark represents the uncertainty regarding the exact mechanism by which it penetrates the peptidoglycan layer after being pumped to the outside [137].

Copper nanoparticles exhibit a broad spectrum of antimicrobial activity against both Gram-positive and Gram-negative bacteria, mediated by multiple and complementary mechanisms, as illustrated in Figure 3 [137]. Several studies have reported stronger activity against Gram-positive bacteria, including *Staphylococcus aureus* and *Bacillus cereus*, compared to Gram-negative species such as *Escherichia coli* and *Pseudomonas aeruginosa*. This difference in susceptibility results from structural features of the bacterial envelopes: The thick but porous peptidoglycan layer in Gram-positive bacteria facilitates the penetration of nanoparticles, while the outer membrane of Gram-negative bacteria acts as an additional barrier [138].

Related to the cell walls, other factors also promote significant influences, such as the chemical composition of the outer membrane, which limits the diffusion of hydrophobic molecules; the presence of efflux pumps, which expel toxic compounds, including antibiotics; the production of specific enzymes, such as β-lactamases; and the expression of resistance genes and mechanisms for adaptation to stress [139].

The antimicrobial effects of CuNPs, widely discussed in this review, especially occur with anisotropic nanostructures such as CuO nanorods [116,140]. For example, CuNPs synthesized by green methods using plant extracts showed inhibition zones of 14–18 mm against *S. aureus* and *B. subtilis*, while they were also effective against *E. coli* and *P. aeruginosa*, demonstrating broad applicability in food safety and medical coatings. Similarly, composites of CuNPs with chitosan films have been successfully tested for food packaging, where they reduced contamination with *Salmonella spp*. and *Listeria monocytogenes* by more than 90% after 24 h [141]. Beyond the direct bactericidal effect, CuNPs embedded in polymers such as chitosan generate biocomposites with applications in medicine, agriculture, and packaging. More recently, studies have extended their use to combat viruses—including SARS-CoV-2—and demonstrated their versatile and cross-domain antimicrobial potential [142].

### 4.2. Antifungal and Antiviral Activities

The antifungal and antiviral potential of CuNPs has gained increasing attention in the face of increasing environmental problems and emerging diseases [143]. CuNPs have been shown to inhibit pathogenic fungi such as *Penicillium digitatum*, *Fusarium* sp., *Alternaria alternata*, *Corticium salmonicolor*, *Aspergillus flavus*, *Aspergillus niger*, *Candida albicans*, and *Phytophthora capsici* [15,144,145]. Practical applications have been reported in the field of post-harvest crop protection: CuNP-based sprays retarded the growth of *Aspergillus* and *Penicillium* on stored fruit and reduced visible spoilage by up to 70%. In medicine, CuNP coatings on dentures significantly reduced the formation of *Candida* biofilms, demonstrating their potential to prevent oral fungal infections. Their antifungal mechanisms include adhesion to cell walls, penetration, and ROS-mediated membrane disruption [145].

Against viruses, CuNPs can bind to capsid proteins or lipid envelopes, promoting irreversible genome degradation—a process described as “kill on contact” [146]. They have demonstrated efficacy against herpes simplex virus-1 (HSV-1), coronavirus (229E), norovirus (MNV-1), infectious avian bronchitis virus (IBV), and bovine herpesvirus (BoHV-1) [48,147]. For example, copper-impregnated face masks were shown to inactivate more than 99% of coronavirus particles within 30 min of contact, highlighting their relevance in PPE during pandemics [148]. Despite promising results, antiviral applications are still relatively under-researched and require improved in vitro/in vivo models to optimize specificity and safety [149]. Beyond biomedicine, the antifungal and antiviral effects of CuNPs position them as potential next-generation agricultural inputs with dual functionality as a crop protection agent and a micronutrient source [150].

### 4.3. Activity Against Multidrug-Resistant Strains

The activity of copper against MDR microorganisms has been highlighted as a non-traditional strategy to combat hospital-acquired infections. In contrast to antibiotics, CuNPs exert their effects through multifactorial mechanisms, as previously discussed, thus reducing the likelihood of cross-resistance [128,151]. Importantly, CuNPs also interfere with biofilm formation, which is central to chronic infections and persistence in clinical settings [127,152].

Clinical evidence shows that surfaces coated with copper alloys in intensive care units lead to a 58% decrease in infection rates, reinforcing the translational potential [127]. In addition, CuO nanoparticles show activity against carbapenem-resistant *Pseudomonas aeruginosa* and *Acinetobacter baumannii*, both of which are classified as critical priority pathogens by the WHO [151]. Further studies have shown that CuNPs inhibit methicillin-resistant *Staphylococcus aureus* (MRSA) biofilms at concentrations as low as 25 µg/mL and significantly reduce bacterial adhesion and matrix formation. In wound healing models, CuNP dressings reduce MDR *E. coli* infection while accelerating tissue regeneration, suggesting a dual therapeutic benefit [153]. At the environmental level, copper supplementation in manure compost reduced the frequency of resistance genes (ermA, ermB) and integrons (intl1) by up to 90%, although selection for copper-specific resistance determinants (copA, cusA) was also observed, highlighting the need for careful management [93]. Applications range from self-disinfecting coatings on high-touch hospital surfaces to copper-impregnated textiles for wound dressings and PPE, expanding the role of CuNPs in MDR control [127,152].

### 4.4. Influence of Nanoparticle Size, Shape, and Concentration on Antimicrobial Efficacy

The antimicrobial efficacy of CuNPs strongly depends on physicochemical parameters. Smaller nanoparticles generally exhibit higher activity because they have a larger surface area to volume ratio, which favors ion release and stronger membrane interactions [154]. For example, CuO NPs of 2 nm showed better bactericidal activity against *E. coli* and *S. aureus* compared to 30 nm analogs [138]. Similarly, 8 nm CuNPs showed significantly larger inhibition zones than 42 nm particles [155].

Morphology also influences activity: anisotropic shapes with sharp edges (e.g., rods, cubes) cause greater membrane disruption than smooth spheres, in some cases leading to a threefold increase in bactericidal efficiency [156]. This profile is due to the fact that different morphologies can alter the mode of action of antimicrobial mechanisms. For example, in relation to ionic release, particles with a large surface area, such as flower-like or nanowires, can release a great amount of copper ions, increasing their toxic effect on the cells [157]. Flower-like nanostructures are considered the most effective form, compared to polyhedral and thumbtack-like nanostructures, suggesting that the difference in the surface free energy may be a cause for their morphology-dependent antimicrobial activity [158].

Shapes with points or edges of this type still promote stronger and more perforated physical contact of the membrane [158]. There is also a relationship with the generation of ROS because morphologies can facilitate the electron transference by Fenton-like reactions [116,140,159]

Concentration plays a dose-dependent role, and concentrations above 60 µg/mL often cause complete inhibition of bacteria, particularly under acidic conditions that enhance ion dissolution [69]. In practical applications, wound dressings loaded with CuNPs at 50 µg/cm^2^ were sufficient to suppress both Gram-positive and Gram-negative colonization in vivo, while higher doses (>100 µg/cm^2^) led to tissue irritation [160].

Regarding the toxicity of copper and CuNP, the applied dose is the main factor. Although higher doses can cause oxidative damage and cytotoxicity, low concentrations can stimulate cellular responses, which might be a problem due to the promotion of undesired antimicrobial-resistant microorganisms. In addition, the dynamic solubility and aggregation of the nanoparticles are a particular issue in determining the actual dose. There is a lack of understanding of chronic exposure and bioaccumulation in in vivo models. In order to provide guidance on the beneficial and toxic doses of CuNPs, we highlight the importance of more comprehensive studies to optimize CuNP design (size, shape, and surface chemistry) and doses to balance antimicrobial efficacy and biosafety.

### 4.5. Copper-Based Nanoparticles in the Inhibition of Quorum Sensing

Microorganisms maintain a biochemical communication among themselves, triggering coordinated actions and expressing collective phenotypes, which can help determine their pathogenicity and survival capacity, bringing a new perspective from a microbiological point of view. This approach has provided promising solutions for issues involving, for example, antimicrobial resistance. This communication system is known as quorum sensing (QS), and it allows interactions between microorganisms after they reach a minimum population density limit, called the critical threshold [161,162].

This phenomenon was first identified in a marine bacterium, *Alivibrio fischeri*. The study found that the bioluminescence phenomenon only occurred when the population density of these bacteria was enough to produce signaling molecules, called autoinducers (AIs). Until they reached a critical threshold (a minimum quorum required) to activate QS, the phenomenon did not occur. Quorum sensing is behind factors that determine, for example, virulence (toxin production), biofilm formation, and consequently antibiotic resistance [162,163,164,165,166].

From this concept, a new therapeutic approach, called quorum quenching (QQ), has emerged. Instead of acting to kill bacteria, as happens with antibiotics, QQ aims to interfere with bacterial communication and consequently prevent them from coordinating the production of factors that lead to the development of virulence or resistance capacity [161,162,166].

Quorum quenching therapy has the potential to reduce selective pressure at non-lethal concentrations, avoiding the direct evolutionary pressure that leads to the rapid development of drug resistance [163,164].

In short, quorum quenching therapeutics act as anti-virulence agents, suppressing the expression of pathogenic phenotypes, such as toxins and biofilm formation (which can create greater resistance). By dismantling these physical and physiological defenses, microorganisms are disarmed, making them vulnerable and weakened to the point that the immune system is able to fight them off naturally or, at least, they are unable to resist traditional antibiotics [164,166,167].

Quorum sensing strategies include the use of enzymes that modify or degrade autoinducers in the extracellular environment, preventing them from reaching the critical threshold necessary to activate quorum sensing. Among the enzymes studied for this purpose, lactonases (AHL-lactonases) and acylases (AHL-acylases) stand out, as well as enzyme inactivation of enzymes that produce signaling molecules. Application of receptor antagonists, or quorum sensing inhibitors, that bind to the receptor and block the gene response without activating it, for example, halogenated furanones, is another option [162,164,167,168].

Metallic nanoparticles, such as copper-based nanoparticles or their nanocomposites, are currently being studied as potential allies in the quorum quenching approach due to their unique physicochemical properties. Studies have reported on the ability of these nanoparticles to inhibit or extinguish quorum detection in microorganisms, as well as to act as anti-biofilm agents, preventing its formation or penetrating and disrupting existing biofilms, thus eliminating the mechanism of bacterial resistance [166,169,170,171,172,173].

## 5. Applications in Biomedical Field

**Figure 4:**
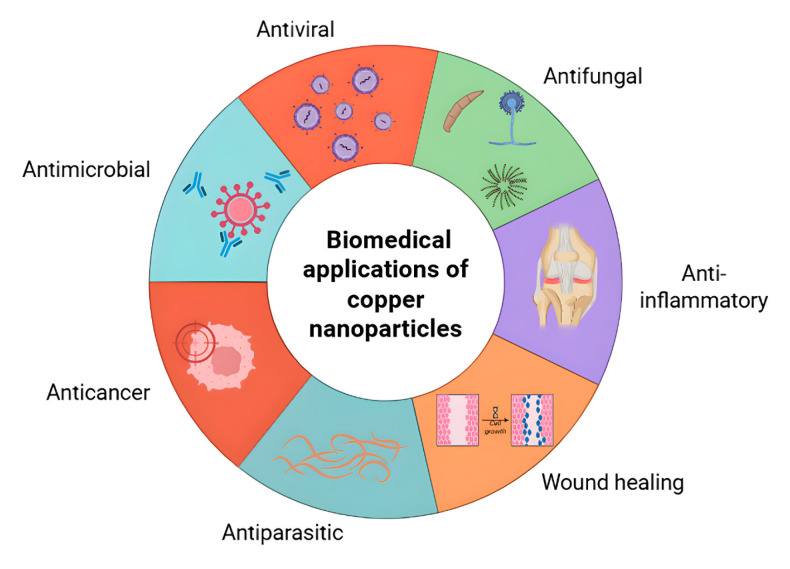
Main biomedical applications of metallic copper nanoparticles (CuNPs). Schematic representation of the most reported and emerging biomedical uses of metallic copper nanoparticles, including antimicrobial, antiviral, antiparasitic, anticancer, anti-inflammatory, and wound-healing activities.

Copper nanoparticles (CuNPs) are increasingly being investigated as multifunctional agents in the biomedical field due to their broad antimicrobial spectrum, their regenerative potential and their compatibility with various materials such as polymers, hydrogels and textiles. Their ability to combine bactericidal effects with additional properties—including osteogenesis, promotion of wound healing, and barrier formation—makes them attractive candidates for next-generation biomaterials. Recent research emphasizes not only their direct antimicrobial role but also their integration into multifunctional systems, in which CuNPs act synergistically with bio-inspired polymers, antioxidants, or nanosensors to achieve higher therapeutic efficacy. Therefore, copper-based nanoparticles are eligible for a broad spectrum of applications in biomedicine, as illustrated in Figure 4.

### 5.1. Incorporation into Medical Devices and Implants

The biomedical applications of copper nanoparticles span several areas, with implants and medical devices being one of the most promising areas. Their primary role lies in antimicrobial coatings, aimed at reducing biofilm formation and implant-associated infections. For example, CuNPs have been investigated as coatings for temporary dental abutments and have shown significant bactericidal activity in both single- and multispecies tests. However, cytotoxicity towards human gingival fibroblasts (HGFs) at low concentrations highlighted the need to optimize biocompatibility, possibly by combining with secondary agents [98]. Similarly, CuNPs incorporated into tissue conditioners for dentures reduced the incidence of denture stomatitis, although further investigation into cytotoxicity and mechanical properties is required [97].

In addition to antimicrobial properties, CuNPs also exhibit osteogenic potential. In guided bone regeneration (GBR), hydrogel membranes enriched with 1 mM CuNPs showed balanced antimicrobial activity, cytocompatibility with HGFs, and osteogenesis in rat mesenchymal stem cells, although validation in vivo is still pending [174]. In the field of reproductive health, copper-based materials are being redesigned into more biocompatible contraceptives. A hydrogel containing CuONPs maintained contraceptive efficacy in rats without causing inflammation or systemic copper accumulation, although long-term safety remains to be determined [175]. Another innovative application involves copper-based nanoenzymes. A copper–heparin–polylysine complex used to coat vascular implants showed anticoagulant effects, improved endothelial regeneration, and led to a 65% reduction in hyperplasia, illustrating the multifunctional potential of CuNPs [176]. Overall, these results suggest that the versatility of copper could enable the development of next-generation implants that combine antimicrobial activity with regenerative and functional properties.

Other developments include orthopedic implants coated with CuNP, which significantly reduced colonization with *Staphylococcus aureus* in rabbit models and at the same time improved osseointegration [https://doi.org/10.1016/j.jmbbm.2024.106674]. In cardiovascular devices, nanostructured copper coatings prevented bacterial colonization of stents and reduced platelet aggregation while exhibiting both antimicrobial and hemocompatible properties [177]. Taken together, these examples highlight the suitability of CuNPs for long-term biomedical use if toxicity management strategies are incorporated into the design.

### 5.2. Use in Wound Dressings and Coatings

Dressings containing CuNPs utilize their potent antimicrobial properties, but the challenge is to balance cytotoxicity with therapeutic benefit. Recent work has focused on multifunctional and sustainable designs. For example, multilayered polymer systems embedded with CuNPs achieved controlled release of nitric oxide (NO) from endogenous donors, reducing bacterial survival by 96.5% while remaining cytocompatible with endothelial cells. However, the stability of the material was limited by structural oxidation [178]. In another study, ZnO/CuO nanocomposites combined with propolis extract accelerated wound closure within six days in vivo and benefited from synergistic antimicrobial and antioxidant effects, although the identification of the metabolites is still ongoing [179]. The production of green nanofibers using lignin to stabilize Ag/CuNPs in polyacrylonitrile (PAN) resulted in antibacterial fibers, although cytotoxicity increased at higher concentrations [69].

More recently, “bio-inspired” approaches have advanced the field of smart dressings. A hydrogel containing catechol molecules and CuNPs mimicked mussel adhesion and exhibited a “sea urchin effect” by puncturing bacterial membranes. This design eliminated 99.9% of bacteria and showed better adhesion to moist skin compared to conventional dressings. Despite the promising results, translation to human trials and cost reduction are still needed [180]. Overall, copper-based nanostructures hold great potential for next-generation wound care solutions. They offer antimicrobial, antioxidant, and bioadhesive properties on a single platform.

Other examples include two-layer hydrocolloid dressings with CuNPs, which not only prevented MRSA infections but also stimulated angiogenesis in diabetic wound models. Another promising development is the incorporation of CuNPs into alginate dressings, which induced a sustained release of copper ions and accelerated epithelialization and collagen deposition in vivo [181]. These multifunctional approaches point to a future in which CuNP dressings serve as both antimicrobial and regenerative platforms.

### 5.3. Integration into Textiles and Packaging Materials

The incorporation of CuNPs into textiles offers antimicrobial protection for applications in healthcare and everyday life. Textiles functionalized with CuNPs have shown activity against bacteria, fungi, and viruses, making them valuable for hospital gowns, face masks, and even sportswear to reduce odor-causing microbes [153,182,183,184]. In packaging, CuNPs improve material flexibility and introduce active barrier functions by reducing oxygen and UV permeability. This extends the shelf life and prevents microbial contamination of foods such as fresh meat, cured meats, cheese, and dairy products, which has a direct impact on reducing spoilage and food safety [185,186,187,188,189]. In addition, CuNPs enable the development of smart packaging systems, in which nanoparticles act as nanosensors capable of detecting chemical or microbial changes and thus monitoring the condition and safety of food in real time [190]. For example, cellulose fibers embedded in CuNP were successfully integrated into surgical masks and achieved >99% inactivation of the influenza virus within 30 min. Similarly, polyethylene films doped with CuNPs reduced *Salmonella enterica* contamination in chicken packaging by over 95%, confirming their role in food safety applications. In sports textiles, CuNP-treated fabrics reduced fungal infections such as athlete’s foot by preventing colonization with *Trichophyton* spp. [141]. These new applications highlight the role of copper not only in infection control but also in the development of sustainable and smart materials in biomedicine, healthcare, and the food industry.

Copper nanoparticles (CuNPs) are increasingly being explored for their broad-spectrum antimicrobial, antiviral, and wound-healing properties. To illustrate the technological landscape and current trends in this field, Table 3 summarizes recent patents (2020–2025) related to the clinical use of copper nanoparticles. These patents encompass applications such as antimicrobial coatings, wound dressings, medical device surfaces, and drug delivery systems, reflecting the translational potential of CuNPs from laboratory research to practical healthcare solutions.

## 6. Toxicity and Impacts

**Figure 5:**
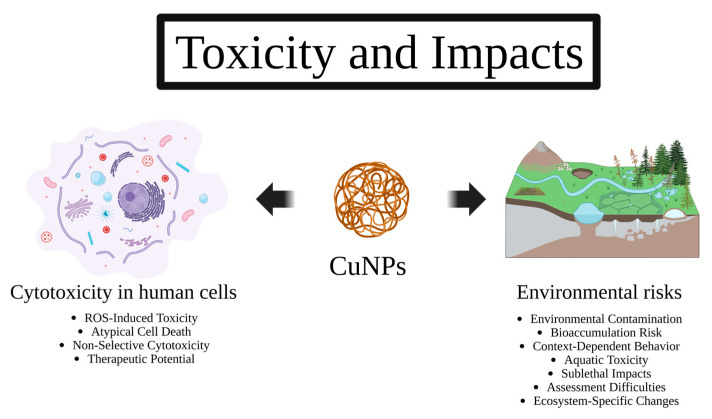
The main cytotoxicity effects of CuNPs in human cells described to date are ROS-induced toxicity, atypical cell death, non-selective cytotoxicity, and therapeutic potential. In terms of environmental impacts, CuNPs can cause contamination, bioaccumulation risk, context-dependent behavior, aquatic toxicity, sublethal impacts, assessment difficulties, and ecosystem changes.

The increasing interest in copper nanoparticles (CuNPs) for biomedical, industrial, and agricultural applications has highlighted the need to carefully evaluate their potential toxic effects. While CuNPs exhibit strong positive points, their interactions with eukaryotic cells, tissues, and environmental systems raise significant safety concerns. Toxicity may result from oxidative stress, cellular dysfunction, or unintended ecological effects, which may limit their practical use (Figure 5). This section provides a comprehensive overview of the cytotoxic and ecotoxicological effects of CuNPs. It highlights both the challenges and opportunities that arise from balancing their therapeutic potential with human and environmental safety. Strategies to mitigate negative effects and improve biocompatibility will also be discussed. Advances in surface engineering, controlled release systems, and predictive toxicology approaches will be highlighted.

### 6.1. Cytotoxic Effects on Eukaryotic Cells and Tissues

As already discussed in this review, one of the most important antimicrobial mechanisms of CuNPs is the induction of reactive oxygen species (ROS). However, it is controversial whether nanoparticles can selectively generate ROS only in the presence of microorganisms. To date, several findings indicate limitations regarding their safe clinical application. For example, a study investigating the cytotoxicity of CuONPs (<10 nm) in both prokaryotic and eukaryotic systems found that eukaryotic cells were more sensitive and experienced cell death at lower concentrations than those required for bacterial inhibition [141]. Compared to soluble copper salts such as CuCl_2_, CuONPs were significantly more toxic and triggered non-apoptotic and non-autophagic pathways of cell death, characterized by mitochondrial dysfunction, glutathione (GSH) depletion, and proteasome inhibition, leading to the accumulation of polyubiquitinated proteins in macrophages [183].

In humans, copper (Cu) functions as a cofactor for fundamental enzymes for metabolism, such as cytochrome C, which is important in cellular respiration, and superoxide dismutase (SOD), an antioxidant enzyme. Primarily absorbed by the duodenum, Cu is transported through the blood associated with chaperones to target organs and can be stored in the liver. When in excess, Cu is mainly released via feces and bile secretion. In cancer, Cu can act as a modulator in cell signaling, stimulating cell proliferation, angiogenesis, and metastasis, both at the transcriptional and substrate levels. In general, cancer cells have disordered Cu regulation with higher demands for the metal than healthy cells. Therapies involving Cu complexes stimulate an increase in intracellular copper concentration in order to cause its cytotoxic effects and decrease its availability in circulation [196].

In vitro studies using two- and three-dimensional models of rat and human intestinal epithelial cells also showed that CuONPs induce higher levels of cytotoxicity and ROS formation than free copper ions (Cu^+^ and Cu^2+^) [96]. Given this cytotoxic potential, some researchers have explored CuNPs as anticancer agents. Experiments on various tumor cell lines (melanoma, breast, and ovary) and on normal fibroblasts exposed to CuONPs, in combination with reducing agents such as N-acetylcysteine (NAC) or ascorbate, confirmed that CuONPs can effectively induce tumor cell death. However, the response was not selective and affected both malignant and healthy cells [197]. Although there are reports suggesting tumor selectivity of CuNPs, the underlying mechanisms remain unclear, and in vivo studies are still needed to validate reproducibility [198]. Recent findings indicate that cytotoxicity is strongly dependent on particle size, surface chemistry and the formation of a protein corona. For example, surface-functionalized CuNPs with polyethylene glycol (PEG) or albumin coatings induced a significantly lower amount of ROS in fibroblasts compared to uncoated CuNPs, suggesting that biocompatibility can be improved by rational surface engineering [199]. In addition, 3D spheroid tumor models showed that functionalized CuNPs exhibited preferential accumulation in hypoxic tumor niches, suggesting that selective cytotoxicity could be achieved when combined with tumor-targeted ligands or stimuli-responsive carriers [200]. Overall, the cytotoxicity of CuNPs towards eukaryotic cells represents both a challenge and an opportunity, highlighting the need for further studies to balance their therapeutic potential with safety concerns.

### 6.2. Ecotoxicological Considerations

In view of the increasing use of copper nanoparticles, it is crucial to assess their potential ecotoxicological impact. Negative consequences include contamination of soil and groundwater, which can affect crops, soil microorganisms, and wider ecosystems through bioaccumulation and trophic transfer [2,25]. CuNPs can enter the environment at different stages of their life cycle (production, transportation, and application) and reach both aquatic and terrestrial systems.

The fate and transport of CuNPs strongly depend on soil type and environmental parameters. For example, sandy soils favor mobility, while loamy and organic-rich soils tend to retain copper. The pH value also influences the stability of the nanoparticles, with higher pH values favoring less soluble copper species [19]. Transformations in the environment, such as redox reactions or interactions with ions and organic material, alter the bioavailability and toxicity of nanoparticles and make ecological risk assessment complex [201]. In biota, copper is an essential trace element but can be toxic at elevated concentrations due to tissue accumulation [202,203]. Biomarkers in aquatic organisms have proven useful to assess the toxicity of CuNPs. Studies have used crayfish, Artemia, Daphnia, mussels, rainbow trout, and other fish species [202,204,205]. Toxicity in fish is of particular concern due to biomagnification in the food chain and can impact both ecosystems and human health [201]. Recent ecotoxicological modeling also shows that CuNPs interact with dissolved organic matter and extracellular polymeric substances in wastewater, forming larger aggregates that may reduce acute toxicity but increase long-term accumulation in sediment. Furthermore, chronic exposure of *Daphnia magna* to low concentrations of CuNPs in mesocosm studies disrupted reproduction rates by up to 40%, even when no acute mortality was observed, emphasizing the importance of sublethal endpoints in environmental risk assessment [206].

Monitoring CuNPs in the environment presents additional challenges: a lack of reliable data on agricultural use, natural background levels of copper in soils, and the lack of standardized ecotoxicological protocols. Variability in nanoparticle size, surface chemistry, exposure to media, and species tested leads to inconsistencies between studies. In marine environments, for example, salt water alters the stability and physicochemical properties of CuNP, leading to different toxicological profiles [207].

### 6.3. Strategies to Mitigate Adverse Effects

Several strategies have been proposed to mitigate the adverse effects of copper nanoparticles. Surface modification is one of the most effective approaches, as it improves the stability of nanoparticles while reducing their undesirable reactivity. The development of stimuli-responsive nanomaterials allows for controlled release in therapeutic contexts, which can increase efficacy while minimizing off-target toxicity. Advanced analytical techniques, including spectroscopy and high-throughput screening, enable better monitoring of biocompatibility and pharmacokinetics. In addition, predictive modeling and computer-aided simulations provide tools for predicting risks and optimizing the design of nanoparticles prior to their application [201].

Recent studies show that hybrid systems combining CuNPs with biodegradable polymers or lipid carriers can significantly reduce systemic toxicity while maintaining antimicrobial efficacy [208]. Stimuli-responsive CuNPs that are activated only under an acidic tumor environment or by external irradiation have also shown promise as a way to minimize collateral tissue damage [209]. Importantly, the implementation of “safe-by-design” approaches that incorporate toxicology data into the early stages of nanoparticle development will be encouraged to accelerate clinical translation without compromising safety.

Finally, the creation of a regulatory framework and standardized risk assessment protocols remains crucial. These should incorporate environmental and clinical perspectives to ensure the safe and sustainable application of CuNPs while enabling innovation in biomedicine and agriculture [210].

## 7. Conclusions and Future Perspectives

Copper nanoparticles (CuNPs) have emerged as one of the most promising alternatives to conventional antimicrobial agents due to their broad spectrum of activity, multiple mechanisms of action, and relatively low cost of production compared to other metallic nanostructures. Their ability to generate reactive oxygen species, disrupt microbial membranes, and interact with DNA and proteins gives them significant efficacy against bacteria, fungi, viruses, and multi-resistant pathogens. Beyond the direct antimicrobial effect, CuNPs have been successfully incorporated into biomedical devices, wound dressings, textiles, and packaging materials, demonstrating their application potential in medicine, agriculture, and the food industry. However, there are still some challenges before widespread clinical and environmental application can be achieved. Cytotoxicity to eukaryotic cells and potential ecotoxicological effects raise biosafety concerns, especially given the tendency of CuNPs to accumulate in tissues and ecosystems. Current evidence suggests that toxicity is highly dependent on particle size, morphology, and surface chemistry, highlighting the need for precise control in synthesis and functionalization. In addition, the lack of standardized test protocols makes reliable risk assessment difficult, hampering comparisons between different studies and limiting regulatory progress.

Future research should focus on strategies that strike a balance between efficacy and safety. Surface modification, green synthesis, and the design of stimuli-responsive nanomaterials are promising ways to improve biocompatibility and reduce adverse effects. Advanced in vitro and in vivo modeling, combined with computer simulations and predictive toxicology, can support the development of safer formulations. From a regulatory perspective, harmonized risk assessment frameworks are urgently needed to establish guidelines that consider both clinical and environmental dimensions. In parallel, the scalability and cost-effectiveness of synthesis methods need to be optimized to ensure that CuNP-based technologies remain accessible. The integration of CuNPs with conventional antibiotics, polymers, and other nanomaterials can also help to reduce the risk of resistance development while improving therapeutic performance. Importantly, the long-term effects of CuNP exposure on patients and ecosystems need to be further investigated to ensure sustainability.

In summary, CuNPs show promise as next-generation antimicrobial agents to address the global challenge of antimicrobial resistance. However, their future success depends on bridging the gap between efficacy in the laboratory and safety in the field. With advances in synthesis methods, toxicity assessment, and regulatory frameworks, copper-based nanomaterials could play a transformative role in biomedical innovation, public health, and sustainable agriculture.

## Figures and Tables

**Table 1:** Differences between different synthesis approaches.

Method	Typical Size Range	Advantages	Disadvantages	Best For	References
Chemical	10–50 nm	Precise control of size and shape; cost-effective; scalable.	Uses hazardous chemicals; requires oxidation prevention.	Applications requiring specific, tunable properties.	[35,43,95]
Physical	2–20 nm	High purity; no chemicals; narrow size distribution.	High cost; low yield; energy-intensive; prone to oxidation.	High-purity applications like electronics (thin films).	[58,65,66]
Biological	20–100 nm	Eco-friendly; biocompatible; uses natural capping agents.	Less control; variable results; slower process.	Biomedical and environmental applications.	[68,71,93]

**Table 2:** Comparison of different metallic nanoparticles with their main advantages and limitations.

Nanoparticle	Advantages	Limitations	References
Copper	Antimicrobial and antifungal activity Promotes wound healing Prevention of food spoilage when used in food packaging Promising catalytic activity	May require organic solvents and high energy input Potential cytotoxicity and environmental impacts May require coating or stabilizing agents	[44,45,96,97,98]
Zinc Oxide	Antimicrobial activity Photocatalytic activity Biosensor activity Plague prevention in agriculture	Potential toxicity Potential pollution	[2,99,100,101]
Silver	Antimicrobial, antifungal, and antiviral activity Anticancer Can be used in water treatment Can act as a catalyst	Expensive Potential toxicity Prone to aggregation/agglomeration	[102,103]
Gold	Drug delivery Biocompatibility Useful for medical imaging Pollution detection	Very high cost Bioaccumulation in certain organs Unpredictable biodistribution in the body	[104,105]
Titanium Dioxide (TiO_2_)	Excellent photocatalytic properties Biocompatibility Low cost Antimicrobial activity	Inactive in dark conditions Potential toxicity	[105,106]

**Table 3:** Recent patents (2020–2025) related to the clinical applications of copper nanoparticles.

Patent Title	Country/ Number	Year	Application	References
Copper metal–organic framework nanoparticle functionalized hydrogel, preparation method, and application thereof	CN112250887-A	2022	Wound healing	[191]
Multilayer copper-based zeolite fiber medical material, protective equipment, and manufacturing method	EP3957341-B1	2024	Antimicrobial medical protection material (e.g., masks and gowns)	[192]
Enhanced antimicrobial efficacy (synergy) of silver and copper compounds and medical use of their combinations	WO2022/224142-A1	2022	Antimicrobial materials, dressings, and formulations	[193]
Fucoidin–copper nanoparticles with antitumor activity, as well as preparation method and application of fucoidin–copper nanoparticles	CN118416098-A	2024	Antitumor activity	[194]
Coatings with antimicrobial copper glass nanoparticles and diol compounds	US11814532-B1	2023	Antimicrobial coating for surfaces	[195]

## Data Availability

No new data were created or analyzed in this study.

## Abbreviations

The following abbreviations are used in this manuscript:

AMR	Antimicrobial resistance
CuNPs	Copper nanoparticles
CuONPs	Copper oxide nanoparticles
*E. coli*	*Escherichia coli*
HGFs	Human Gingival Fibroblasts
MDR	Multidrug Resistance
NAC	N-acetylcysteine
NO	Nitric oxide
PAN	Polyacrylonitrile
PEG	Polyethylene glycol
PVD	Physical vapor deposition
PVP	Polyvinylpyrrolidone
ROS	Reactive Oxygen Species
*S. aureus*	*Staphylococcus aureus*
WHO	World Health Organization
